# The intergenerational impact of war on mental health and psychosocial wellbeing: lessons from the longitudinal study of war-affected youth in Sierra Leone

**DOI:** 10.1186/s13031-020-00308-7

**Published:** 2020-09-01

**Authors:** Theresa S. Betancourt, Katrina Keegan, Jordan Farrar, Robert T. Brennan

**Affiliations:** 1 Research Program on Children and Adversity, Boston College School of Social Work, Chestnut Hill, MA USA; 2 Women’s Studies Research Center, Brandeis University, Waltham, MA USA

**Keywords:** Implementation science, Post-conflict, Ebola virus disease, Ethics, Humanitarian crisis, Child soldiers, Intergenerational trauma, Global adversity, Longitudinal research, Risk and protective factors

## Abstract

**Background:**

Globally, one in four children lives in a country affected by armed conflict or disaster often accompanied by exposure to a range of adversities including violent trauma and loss. Children involved with armed groups (often referred to as “child soldiers”) typically exhibit high levels of mental health needs linked to their experiences. The Longitudinal Study of War-Affected Youth (LSWAY) in Sierra Leone is a seventeen-year prospective longitudinal study of the long-term effects of children’s experiences in the country’s eleven-year (1991–2002) civil war on their adult mental health and functioning in addition to exploring the potential mechanisms by which intergenerational transmission of emotional and behavioral disruptions due to war trauma may operate. LSWAY illuminates how war-related and post-conflict experiences shape long-term adult functioning, family dynamics, and developmental outcomes in offspring.

**Discussion:**

The LSWAY study utilizes mixed methodologies that incorporate qualitative and quantitative data to unpack risk and protective factors involved in social reintegration, psychosocial adjustment, parenting, and interpersonal relationships. To date, study findings demonstrate striking levels of persistent mental health problems among former child soldiers as adults with consequences for their families, but also risk and protective patterns that involve family- and community-level factors. This case study examines the course of LSWAY from inception through implementation and dissemination, including building on the study results to design and evaluate several intervention models.

**Conclusion:**

The case study offers a unique perspective on challenges and field realities of health research in a fragile, post-conflict setting common in the context of humanitarian emergencies. LSWAY findings along with lessons learned from the field can inform future research as well as intervention research and implementation science to address the mental health and development of war-affected young people. With four waves of data collection and a planned fifth wave, LSWAY also provides rare insights into the intergenerational effects of humanitarian crises on children, youth, and families across generations.

## Background

### Humanitarian context

One in four children lives in a country affected by conflict or disaster [1]. War-affected children often face adversities such as forced family separation, loss of access to school and healthcare, insecure access to food and shelter, and displacement from homes and communities [2]. In some of the world’s most violent armed conflicts, the exploitation of children by armed groups has increased in recent years [3]. Children involved with armed groups are at heightened risk for mental health problems including depression, anxiety, and posttraumatic stress disorder (PTSD) [4–6]. Former child soldiers often face stigma and lack family and community acceptance upon returning home [7].

Like many of today’s West African countries, Sierra Leone was exploited during the transatlantic slave trade. During the American Revolutionary War, the British offered freedom to slaves who supported their cause. Following their outlawing of slavery in 1807, the British brought captives from captured slaves ships to Sierra Leone, which became a British Colony and Protectorate in 1808, gaining its independence in 1961. Eleven years of civil war (1991–2002) left Sierra Leone with weakened community structures, insufficient social services and dramatically under-functioning systems of education and healthcare. The Ebola virus disease (EVD) outbreak from 2014 to 2015 and mudslides from 2017 wreaked further havoc.

Limited mental health care personnel, inadequate funding for mental health interventions and treatment, and stigma of mental health problems present significant barriers to accessing care in Sierra Leone. The effects of trauma from living through conflict, epidemics, and natural disasters are long-lasting and may be transmitted intergenerationally [8, 9].

### Research study

The Longitudinal Study of War-Affected Youth (LSWAY) is a prospective longitudinal intergenerational study of male and female former child soldiers who participated in Sierra Leone’s civil war. The study began following the end of conflict in 2002 with the goal of illuminating risk and protective factors shaping social reintegration and psychosocial adjustment over time. As the cohort has aged into young adulthood, LSWAY data have contributed to understanding how war experiences and post-conflict hardships impact adult functioning, family dynamics such as parenting and intimate partner relationships, and developmental outcomes in offspring.

**Fig. 1:**
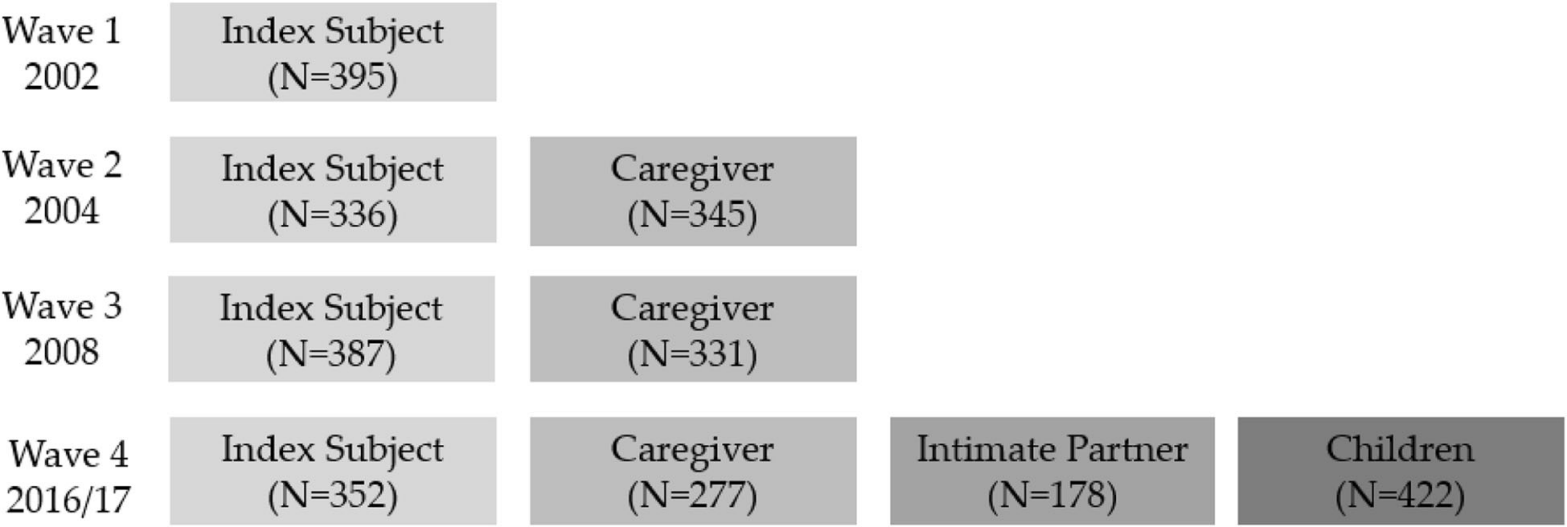
Research design and respondents by wave of data collection

LSWAY began as a collaboration between researchers and a large international nongovernment organization (NGO). Surveys were developed in collaboration with local NGOs and community advisory boards (CABs). Data were collected at four time points (T1: 2002, T2: 2004, T3: 2008, T4: 2016/2017) determined by funding and access dynamics (Fig. 1). At baseline, subjects aged 10–17 who were involved with the Revolutionary United Front (RUF) or other armed groups, who had been referred to Disarmament, Demobilization, and Reintegration (DDR) programs and were on the roster of a collaborating interim care center serving five districts (Bo, Kenema, Kono, Moyamba, and Pujehun) were invited to participate in the study (*n* = 259). A random door-to-door survey of youth of similar age not served by DDR programs (*n* = 136) was administered in the same five regions. Additional self-reintegrated youth were added at T2 from an NGO outreach list from the Makeni region which was the last to be released from rebel control (*n* = 127). The cohort’s caregivers were included from T2 onward and their intimate partners and children were added at T4.

All participants were interviewed in Sierra Leone Krio, the most widely spoken language, by trained Sierra Leonean research assistants (RAs). Basic demographic information was collected as well as length of involvement with armed groups, war-related violence exposures, and standardized scales of psychosocial adjustment developed and validated for use among former child soldiers in Sierra Leone [10]. The survey also included questions about family configuration and relationships, community acceptance, social support, access to educational and skills-training opportunities, and family socioeconomic status upon return. Follow-up surveys at all waves repeated these baseline measures. At T2, measures were added to examine social capital, stigma/discrimination, high-risk behavior, civic participation, post-conflict hardships, and a more detailed inventory of war trauma. At T4 89% (*n* = 352) of the sample of former child soldiers were reassessed. At T4, intimate partner violence, parenting and child well-being measures were added. See Additional file 1 for a list of measures used with index subjects and their offspring.

Most of the sample was forced into the RUF (87%, *n* = 460; male = 354, female = 106) at a mean age of 10.9 years and remained an average of 3.3 years. Twenty-five percent (*n* = 131) reported killing or injuring others (male = 104, female = 27), 71% (*n* = 377) reported witnessing violence (male = 281, female = 96), and 66% (*n* = 351) reported having been a victim of violence (male = 89, female = 262) during the war. Ten percent (*n* = 51) reported being raped during the war (male = 15, female = 36) and 23% (*n* = 123) reported having a parent die during the war (male = 86, female = 37).

Long-term mental health outcomes of former child soldiers were shaped by war experiences and post-conflict factors [11–14]. Lower levels of prosocial behavior were associated with having killed or injured others during wartime and with experiencing social stigma after the war. Those who reported having been raped exhibited heightened post-war anxiety and depression. Worsening anxiety and depression over time were also closely related to being involved in fighting forces at a younger age and to post-conflict social and economic hardships.

At T4, 47% exceeded the threshold for anxiety/depression and 28% exceeded the likely PTSD threshold [14]. Using latent class trajectory modeling, three groups were identified based on changes in stigma and family/community acceptance; “Improving Social Integration” fared nearly as well as the “Socially Protected.” The “Socially Vulnerable” group, who experienced the highest sustained stigma and lowest community and family acceptance, had increased risk of anxiety/depression above the clinical threshold, possible PTSD, and were around three times more likely to attempt suicide.

Initial analyses of the intergenerational data indicate correlations between parental emotion dysregulation and that of offspring. A future fifth wave of data collection proposes to investigate the mechanisms by which intergenerational effects may arise including exploration of clinical and biological data that may be collected ethically and with cultural sensitivity as well as collection of observational assessments to push beyond the limitations of self-report survey data.

## Discussion

### Scientific importance of this research

Research on war-affected populations is often cross-sectional which limits insight into how former child soldiers fare as adults. More mixed-method work is needed to understand what resilience and healthy social functioning look like in varied contexts and cultures [15]. Finally, most research on former child soldiers has focused on exposure to war-related violence without exploring how post-conflict factors, such as ongoing hardships, stigma, and family and community acceptance influence long-term adjustment and life outcomes [14].

LSWAY addresses these gaps and is the first longitudinal/intergenerational research in sub-Saharan Africa to examine the role of post-conflict factors in shaping the mental health and social reintegration of male and female former child soldiers over time [16, 17]. The research has helped to advance a mixed-methods approach to understanding local terms for mental health related problems [18] and protective processes and resources that might be leveraged by intervention models. LSWAY findings have also informed the development of evidence-based interventions, including the Youth Readiness Intervention (YRI), a group mental health treatment with demonstrated effectiveness for addressing emotion dysregulation, interpersonal skills, and improving functioning among war-affected youth [19–21] and later Youth FORWARD (NIMH 1U19MH095705–01), a research collaboration to scale out evidence-based mental health interventions for youth exposed to adversities in West Africa. Although the context of humanitarian emergencies presents formidable challenges for conducting ethical and rigorous research, the challenges of such environments can also advance innovative problem solving that has implications for other low-resource settings, including those not affected by emergencies (Table 1). Innovations in adapting and selecting tools and using mixed-methods to communicate mental health conditions in less stigmatizing terminology is an approach that can be applied to diverse cultural settings. In addition, the relative paucity of highly trained mental health professionals in Sierra Leone requires intervention models that can be delivered by lay workers who receive robust training under targeted supervision structures.

**Table 1:** Summary of research challenges and strategies in humanitarian contexts

Challenge		Strategies
Ethical	Dismantling power differentials between researchers and vulnerable populations	▪CBPR to ensure community engagement, co-learning, and involvement
		▪Community advisory boards to reinforce and enrich investigation
	Ensuring appropriateness, sensitivity and relevance in local contexts	▪Employ an ecological approach to investigate individual, family, and community influences
		▪Mixed-methods to establish culturally meaningful and valid assessments
		▪Focus group discussions to refine items and determine cultural appropriateness
		▪Develop locally derived measures in close consultation with local staff and community members
	Addressing risk of harm cases	▪Anticipate risk of harm cases and develop rigorous protocols to ensure participant safety, appropriate referrals, and follow-up
Capacity/Sustainability	Developing long-term, stable partnerships	▪Develop a capacity-building core to develop and deliver innovative, locally relevant training and technical assistance programs
	Establishing systems of follow-up	
	Supporting rather than overburdening professionals	
Logistical	Adapting to unforeseen, adverse circumstances	▪Anticipate and plan for them. When that fails, accept the challenge presented, make appropriate adaptations to the research process and consider opportunities
Conceptualizations and Assessment of Mental Health in Diverse Cultures and Contexts	Assessing mental health problems, especially in the absence of validated screening tools	▪Involve local teams in qualitative data collection to understand stigma and co- morbidity and to examine protective processes linked to resilient outcomes
	Addressing stigma around mental health	▪Use mixed methods models to select, adapt, or create measures of mental health and related constructs
	Addressing co-morbidity	
		▪Use psychometric methods to subject tools to rigorous validation, testing and refinement

### Conducting research in humanitarian settings

#### Ethical challenges

When conducting research in humanitarian settings, it is important to recognize the power differential that exists between researchers and vulnerable populations. These dynamics must be carefully considered, and methodologies developed to ensure all stakeholders are engaged in equitable, ethical, and effective collaboration. LSWAY utilized concepts from community-based participatory research (CBPR) to ensure community engagement and study oversight [22–25]. A commitment to CBPR methods meant the involvement of both community and academic partners in designing and implementing the research. CABs, comprising various stakeholder groups, were established to reinforce and enrich investigation.

Ethical challenges are inherent in working across cultures and a concerted effort is needed to ensure appropriateness, sensitivity, and relevance of research within the local context. LSWAY’s scientific methods are informed by an ecological approach, which investigates individual, family, and community influences on child health and well-being [26]. We used mixed-methods to establish culturally meaningful and valid assessments of mental health, risk and protective factors, and social functioning [27, 28]. Surveys contained a mix of standard and locally-derived measures, adapted and developed in close consultation with local staff and community members (Additional file 1). Focus group discussions among youth and adults similar to the study population helped refine items and determine cultural appropriateness of scales.

Another ethical challenge is the potential for risk of harm cases. Such instances include risk of suicide, risk of physical or sexual abuse, risk of harming another person, or risk of being a victim of crime. Researchers must be prepared to respond should risk of harm cases arise; no easy feat in humanitarian settings where access to mental health treatment and social welfare services is often limited or nonexistent. Contextually grounded protocols provided study staff with structured approaches to address high-risk situations, while ensuring participant safety, appropriate referrals, and follow-up. RAs also received robust training on supporting participants as they recounted trauma experiences, often through roles plays and with input from CABs. Procedures included providing breaks as needed and bringing in study mental health workers when necessary. Those considered a risk to themselves or others or at risk of immediate harm due to abuse and neglect were referred for appropriate mental health care, social work services, and/or other appropriate authorities. Researchers dedicated seven months to prepare mechanisms and identify formal and non-formal supports to help anticipate, identify, and address risk of harm cases [11, 16]. We considered it critical to anticipate and plan for referrals and to consider resources outside formal services. Local village leaders were instrumental to ensure safety and well-being of participants at risk of harm.

Another major ongoing ethical concern is protecting subjects’ identities and the confidentiality of their data. Data security overall is an issue of importance when working in the context of sensitive topics. Study protocols require that all data are deidentified and linked only by unique ID numbers, and always stored separate from identifying information. Data are stored in encrypted formats, in password-protected cloud-based servers and all collaborators are asked to participate in weekly data review meetings and to abide by agreements on data use and ethics.

#### Capacity/sustainability challenges

When LSWAY began in 2002, many NGOs were operating in the region to serve war-affected youth. However, as the country transitioned from a crisis to a fragile state, many organizations discontinued operations, presenting challenges to developing stable, long-term partnerships and establishing systems of follow-up essential for longitudinal research. The paucity of highly-trained professionals, their limited bandwidth given competing priorities, and limited funding to cover their efforts is another major challenge in Sierra Leone that requires strategies to support the few individuals qualified and interested in building research capacity. Technological barriers including poor cell phone coverage, inconsistent internet connection, and frequent power outages also limit access to capacity building opportunities such as webinars delivered by out-of-country experts.

As one of six goals identified in the Grand Challenges in Global Mental Health Initiative led by the National Institute of Mental Health and the Global Alliance for Chronic Disease [29], capacity building has been recognized as a specific approach to closing the mental health treatment gap and transforming the lives of those individuals affected by armed conflict. Within Youth FORWARD, a primary aim is the development of a capacity building core that will develop and deliver locally relevant training and technical assistance programs to (a) increase the regional knowledge base and capacity for implementation research in mental health, (b) increase the use of science-based methods for developing mental health policies and programs, (c) increase the use of rigorous evaluation methods to measure program effectiveness, and (d) accelerate efforts to scale up evidence-based mental health programs to close the mental health treatment gap.

#### Logistical challenges

Even when anticipating and planning for challenges, unforeseen circumstances often arise throughout the research process. This is especially true of research in humanitarian settings and complex emergencies where volatile and precarious conditions are commonplace. Some challenges require careful consideration and innovative adaptation to the research strategy, partnerships, or resources. Other challenges require patience and problem solving in the face of major study interruptions and delays.

Some unforeseen challenges can be overcome with fairly simple adaptations, the delaying of timelines, or the shifting of resources. Following the death of the director of the collaborating NGO, researchers were forced to terminate the data collection at T2 after reaching only 56.5% of the original sample. To account for this abrupt change, the team re-interviewed 68.8% of the original sample at T3, including some who were not re-interviewed at T2. Thus, the research team accepted the challenge presented and made appropriate adaptations to the research process to address it.

Some challenges, however, carry more devastating disruptions and require creative responses. In the wake of the EVD crisis, data collection was suspended at T4 with NIH approval, and the Principal Investigator began fundraising and making plans to address the responses and psychosocial triggers of the outbreak in a separate study. Despite operating in a state of emergency, researchers drew from prior strengths and long-standing LSWAY partnerships and deployed a local team in Western Sierra Leone. Three waves of data collection assessed community knowledge, attitudes, and behaviors via surveys, and formative qualitative research to examine how war-related trauma and/or past mental health problems affected residents’ responses to health messaging and their engagement in both risky and health-promoting behaviors [26].

Research examined associations between war exposures, PTSD symptoms, depression, anxiety, personal EVD exposure, and related behaviors among *N* = 1008 adults (98% response rate) randomly sampled at the height of the epidemic (January–April 2015). Findings demonstrated that EVD risk behaviors were associated with depression and PTSD symptoms while EVD prevention behaviors were associated with higher anxiety, having a friend diagnosed with EVD, and higher previous war exposure, independent of mental health (see Additional file 2). While this study is specific to the Western Area of Sierra Leone, its implications are important for other countries affected by conflict and EVD, such as the Democratic Republic of the Congo, currently home of the second largest EVD outbreak to date as well as the many regions around the world affected by the global COVID-19 outbreak.

#### Conceptualizations and assessment of mental health in diverse cultures and contexts

In the field of Global Mental health, there are challenges to assessing and determining levels of mental health problems as oftentimes areas affected by humanitarian disasters do not have readily available validated tools for screening mental health problems and providing reliable and valid psychometric properties making it possible to assess program effectiveness. In addition, the concept of disorder or pathology when many members of society are impacted may be stigmatizing or disempowering. In the context of complex trauma, the co-occurrence of mental health conditions such as depression and post-traumatic stress reactions, is common, making both assessment and intervention planning challenging.

### Research strategies

#### Strategies to address challenges in conceptualizing and assessing mental health in diverse cultures and contexts

**Fig. 2:**
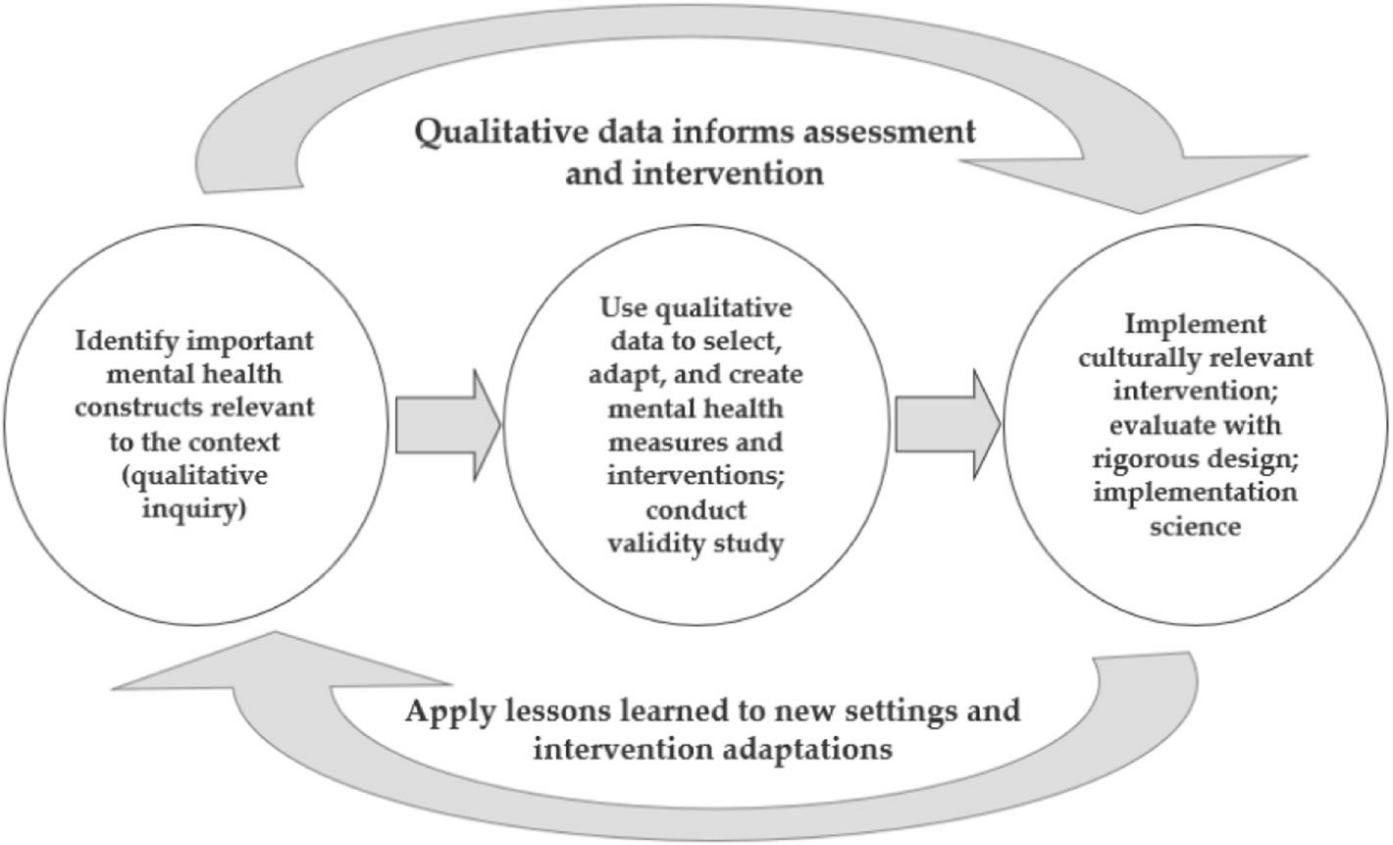
Model for designing and evaluating mental health services in diverse cultural settings

Our research team has built out models that apply a sequence of qualitative and quantitative methods to address some of the measurement challenges faced by the field (Fig. 2). We often collect qualitative data wherever possible early and with rapid approaches involving local RAs who speak both English and the local language and use this qualitative data to select, adapt, or create measures of mental health and related constructs and to subject these to rigorous quantitative validation tests and refinement via psychometric methods [30–32]. Similar approaches can be used to examine local protective processes linked to resilient outcomes that can become the “active ingredients” of strengths-based interventions.

#### Lessons learned

In conducting mental health research with vulnerable populations, the research team should anticipate coming into contact with a significant number of vulnerable participants who require responses to ensure participant safety with referrals to higher levels of care. This is particularly true in research with vulnerable populations where participants are often asked to share sensitive and personal information but live in settings where access to mental health services is poor [33–36]. Anticipation of and planning in response to risk of harm cases before the study begins is an essential part of conducting research with vulnerable groups.

Another challenge regards sustainability and capacity. The CBPR process can ensure long-term, equitable partnerships are established, but capacity building efforts are needed in order to reduce the burden. For example, partnerships with community-based mental health professionals are vital for ensuring risk of harm cases are appropriately addressed. At the same time, however, researchers must understand the needs and realities of such systems of care. As such, capacity building efforts must be embedded in the partnership to ensure that mental health professionals on the ground are provided the opportunity to gain needed skills, participate in dissemination activities, and contribute to the policy-making process.

LSWAY and the process of working in a fragile setting like Sierra Leone has also highlighted the logistical challenges of this work. While some challenges can be anticipated and planned for, like risk of harm scenarios, others are unforeseen, like the EVD epidemic. It is crucial that investigators and their in-country teams are flexible and adaptable, and engage in creative problem solving at all stages of the research process. Strong communication channels and partnerships are especially vital to this process with researchers being comfortable using a variety of communication modalities that are responsive to bandwidth and connectivity issues inherent in post-conflict settings.

Moving from observational to intervention research yields several insights. First, there is a need to move beyond a focus on past trauma and war experiences to address post-conflict factors and understand factors shaping risk and resilience. We advocate for greater investment in strengthening services to promote family and community acceptance, reduce stigma, and expand social supports. Our findings indicate that youth can positively affect their own family and community reintegration through self-regulation, prosocial behavior, and interpersonal skills, which are often disrupted by trauma exposure. Accordingly, targeted interventions can adapt evidence-based practices common in mental health interventions to enhance self-regulation, functioning, and interpersonal skills to support reintegration. Second, services should target those most in need-based on current assessments of distress and impairment, rather than durable “labels” that may lead to an increase in societal stigma for already vulnerable youth. Sustainable national and community-level systems are needed that respond to the social service needs of *all* youth and families that experience ongoing difficulties due to co-occurring forms of adversity inherent in war affected settings [37]. Finally, implementation science approaches can be applied to scaling up and scaling out [38] relevant evidence-based interventions to strengthen social services while recognizing the need to adapt to the evolving needs of individuals and families.

## Conclusions

War-affected youth live with exposure to violence, loss, and separation during armed conflict as well as ongoing hardships. Contextual and individual factors influence whether such youth successfully overcome the effects of these traumas. Enhancing understanding of such factors can improve efforts to promote resilience in war-affected youth and target attention to youth most in need of psychosocial support. Given the current spread of armed conflict and disaster across many regions of the world, and the exposure of children and youth to adversity, it is imperative to understand the drivers of resilience in the aftermath of humanitarian crises and the long-term effects of compound adversity, so that those both directly and indirectly affected can resume positive life trajectories and reach their fullest developmental potential.

## Supplementary information

**Additional file 1.** LSWAY Measures.

**Additional file 2.** EVD Measures.

## Data Availability

Not applicable. If your manuscript does not contain any data, please state ‘Not applicable’ in this section.

## Abbreviations

CAB: Community Advisory Board
CBPR: Community-Based Participatory Research
DDR: Disarmament, Demobilization, and Reintegration
EVD: Ebola Virus Disease
LSWAY: Longitudinal Study of War-Affected Youth
NGO: Non-Governmental Organization
PTSD: Posttraumatic Stress Disorder
RA: Research Assistant
RUF: Revolutionary United Front
