# Supplementary material for: The intergenerational impact of war on mental health and psychosocial wellbeing: lessons from the longitudinal study of war-affected youth in Sierra Leone
Source: Confl Health. 2020 Sep 1;14:62. doi: 10.1186/s13031-020-00308-7 (PMC7461150; doi:10.1186/s13031-020-00308-7)
Supplement: Supplementary file 1 — Additional file 1. LSWAY Measures. [file 13031_2020_308_MOESM1_ESM.docx]

**Appendix A. LSWAY Measures**

| **Index Subject/Former child soldiers measures** | |
| --- | --- |
| **Measures** | **References** |
| Sampson’s Collective Efficacy Scale | Sampson, R.J., Raudenbush, S.W., & Earls, F. (1997). Neighborhoods and violent crime: a multilevel study of collective efficacy. *Science*, 277 (5328), 918-924. |
| Hopkins Symptom Checklist (HSCL) | Derogatis, L.R., Lipman, R.S., Rickels, K., Uhlenhuth, E.H., & Covi, L. (1974). The Hopkins Symptom Checklist (HSCL): a self-report symptom inventory. *Behavioral Science,*19(1), 1-15. |
| Everyday Discrimination Scale | Williams, D.R., Yu, Y., Jackson, J.S., Anderson, N.B. (1997). Racial differences in physical & mental health: Socioeconomic status, stress, and discrimination. *Journal of Health Psychology,* 2(3), 335-351.  Schulz, A. J., Israel, B.A., Zenk, S.N., et al. (2006). Psychosocial stress and social support as mediators of relationships between income, length of residence and depressive symptoms among African American women on Detroit's eastside. *Social Science & Medicine,* 62(2), 510-522.  Siefert, K., Williams, D. R., Finlayson, T. L., Delva, J., & Ismail, A.I. (2007). Modifiable Risk and Protective Factors for Depressive Symptoms in Low-Income African American Mothers. *American Journal of Orthopsychiatry,*77(1), 113-123. |
| Scale of Perceived Community Acceptance | Betancourt, T.S., Agnew-Blais, J., Gilman, S.E., Williams, D.R., & Ellis, B.H. (2010). Past horrors, present struggles: the  role of stigma in the association between war experiences and psychosocial adjustment among former child soldiers in Sierra Leone. *Social Science & Medicine,*70(1),17-26. |
| Oxford Measure of Psychosocial Adjustment (OMPA) | MacMullin, C., & Loughry, M. (2004). Investigating psychosocial adjustment of former child soldiers in Sierra Leone and Uganda. *Journal of Refugee Studies,*17(4), 460-472. |
| Post Traumatic Stress Disorder Reaction Index (PTSD-RI) | Steinberg, A. M., Brymer, M. J., Decker, K. B., & Pynoos, R. S. (2004). The University of California at Los Angeles post-traumatic stress disorder reaction index. Current psychiatry reports, 6(2), 96-100. |
| Post-Traumatic Growth Inventory (PTGI) | Tedeschi, R. G. & Calhoun, L. G. (1996), The posttraumatic growth inventory: Measuring the positive legacy of trauma. J. Traum. Stress, 9: 455–471. |
| Post-War Adversities Scale | Layne, C.M., Stuvland, R., Saltzman, W., Djapo, N., & Pynoos, R.S. (1999). *Adolescent Post War Adversities Scale*: Unpublished instrument. |
| Forgiveness Scale | Toussaint, L. L., Williams, D. R., Musick, M. A., & Everson, S. A. (2001). Forgiveness and health: Age differences in a US probability sample. *Journal of adult development*, *8*(4), 249-257. |
| Difficulties in Emotion Regulation Scale (DERS) | Gratz, K., & Roemer, L. (2004). Multidimensional Assessment of Emotion Regulation and Dysregulation: Development, Factor Structure, and Initial Validation of the Difficulties in Emotion Regulation Scale. *Journal of Psychopathology and Behavioral Assessment,* 26(1), 41-54. |
| Brief COPE Scale | Carver, C. (1997). You want to measure coping but your protocol’s too long: Consider the brief cope. *International Journal of Behavioral Medicine,* 4(1), 92-100. |
| Brief RCOPE (religious coping) | Pargament, K., Feuille, M., & Burdzy, D. (2011). The Brief RCOPE: Current psychometric status of a short measure of religious coping. *Religions*, *2*(1), 51-76.  Annan, J., Blattman, C., Carlson, K., & Mazurana, D. (2007). *The Survey of War Affected Youth: Making reintegration*  *work for youth in northern Uganda* 2007.  Annan, J., Blattman, C., Carlson, K., & Mazurana, D. (2005). Survey of War-Affected Youth. |
| Measure of Functional Limitations | Adaptation of WHO – Disability Adjustment Scale to Sierra Leone based on qualitative data.  World Health Organization (WHO). WHO Disability Assessment Schedule (WHO-DAS) 2.0. 2010. |
| WHO-Disability Adjustment Scale | World Health Organization (WHO). WHO Disability Assessment Schedule (WHO-DAS) 2.0. 2010. |
| SWAY Parenting | Annan, J., Blattman, C., Carlson, K., & Mazurana, D. (2007). *The Survey of War Affected Youth: Making reintegration work for youth in northern Uganda* 2007.  Annan, J., Blattman, C., Carlson, K., & Mazurana, D. (2005). Survey of War-Affected Youth. |
| Dyadic Adjustment Scale (DAS) | Spanier, G. (1976). Measuring Dyadic Adjustment: New Scales for Assessing the Quality of Marriage and Similar Dyads. *Journal of Marriage and Family,* *38*(1), 15-28. |
| Conflict Tactics Scales | Straus, M.A.(1979). Measuring Intrafamily Conflict and Violence: The Conflict Tactics (CT) Scales. *Journal of Marriage and Family,* 41(1), 75-88.  Moffitt, T.E., Caspi, A., Krueger, R.F., et al. (1997). Do partners agree about abuse in their relationship?: A psychometric evaluation of interpartner agreement. *Psychological Assessment,* 9(1), 47-56. |
| *Adapted* Youth Risk Behavior Survey | Adu-Mireku, S. (2003). Family communication about HIV/AIDS and sexual behaviour among senior secondary school students in Accra, Ghana. *African Health Sciences*, 3(1), 7-14.  Center for Disease Control. State and Local Youth Risk Behavior Survey. 2005; http://www.cdc.gov/HealthyYouth/yrbs/pdfs/2005highschoolquestionaire.pdf. Accessed October 30, 2006.  Centers for Disease Control and Prevention. YRBSS: Youth Risk Behavior Surveillance System. Atlanta, GA: Centers for Disease Control and Prevention 1991. |
| EVD Hardships and Daily Hassles | Adapted to EVD based on the Post War Adversities Scale:  Layne, C.M., Stuvland, R., Saltzman, W., Djapo, N., & Pynoos, R.S. (1999). *Adolescent Post War Adversities Scale*: Unpublished instrument. |
| Beliefs about Ebola | Duncan, L. A., Schaller, M., & Park, J. H. (2009). Perceived vulnerability to disease: Development and validation of a 15-item self-report instrument. *Personality and Individual differences*, *47*(6), 541-546. |

| **Offspring Measures** | | | |
| --- | --- | --- | --- |
| **Measures** | **Age (year)** | **Reporter** | **References** |
| Home Observation For Measurement of the Home Environment (HOME-Uganda) | 0-2.9 | Observer | Totsika, V., & Sylva, K. (2004). The Home Observation for Measurement of the Environment Revisited, 9, 25-35. |
| Observation of Mother-Child Interaction (OMCI) | 0-2.9 | Observer | Rasheed, M.A., & Yousafzai, A.K. (2013 September). The development and reliability testing of an observation tool for assessing mother-child interaction in field studies- Experience from Pakistan In: The 16th European Conference on Developmental Psychology, Lausanne, Switzerland |
| Malawi Development Assessment Tool (MDAT) | 0-2.9 | Observer | Gladstone, M., Lancaster, G., Umar, E., et al. (2010). The Malawi Development Assessment Tool (MDAT): The Creation, Validation and Reliability of a Tool to Assess Child Development in Rural African Settings. *PLoS Med,* 7(5), 1-14 |
| Preschool Self-Regulation Assessment (PSRA) | 0-2.9 | Observer | The PSRA assessor report draws from previous work capturing assessors' global readings of children's regulation (Roid & Miller, 1997; Wakschlag et al., 2005) |
| Parental Acceptance and Rejection Questionnaire Short Form (PARQ) | 0-2.9 | Caregiver | Rohner, R. P., & Khaleque, A. (Eds.). (2005). Handbook for the Study of Parental Acceptance and Rejection (4th ed). Storrs, CT: Rohner Research Publications. |
| Adapted UNICEF Multiple Indicator Cluster Survey (MICS) | 0-2.9 | Caregiver | UNICEF. Child Disciplinary Practices at Home. Retrieved July 16, 2020, from <https://www.unicef.org/protection/Child_Disciplinary_Practices_at_Home.pdf> |
| Ages and Stages Questionnaire (ASQ) | 0-2.9 | Caregiver | American Academy of Pediatrics. (2001). Developmental Surveillance and Screening of Infants and Young Children. *Pediatrics, 108*(1), 192–196.  American Academy of Pediatrics. (2006). Identifying Infants and Young Children with Developmental Disorders in the Medical Home: An Algorithm for Developmental Surveillance and Screening. *Pediatrics, 118*, 405–420.  Beam, M., Paré, E., Schellenbach, C., Kaiser, A., Murphy, M., (2015). Early Developmental Screeinng in High-Risk Communities: Implications for Research and Child Welfare Policy. *The Advanced Generalist: Social Work Research Journal, 1(3/4),* 18-3  Hanig, K. M. (2010). Review of Ages & Stages Questionnaires®: A Parent-Completed Child Monitoring System. In R. A. Spies, J. F. Carlson, & K. F. Geisinger (Eds.), *The eighteenth mental measurements yearbook,* 10–13. Lincoln, NE: Buros Institute of Mental Measurements.  Squires, J., Bricker, D., & Potter, L. (1997). Revision of a parent-completed developmental screening tool: Ages and Stages Questionnaires. *Journal of pediatric psychology*, *22*(3), 313-328. |
| Ages and Stages Questionnaire Social and Emotional (ASQ - SE) | 0-2.9 | Caregiver | Squires, J., Bricker, D., & Twombly, E. (2002). *The ASQ:SE user’s guide.* Baltimore, MD: Paul H. Brookes Publishing Co.  Squires, J., Bricker, D., & Potter, L. (1997). Revision of a parent-completed developmental screening tool: Ages and Stages Questionnaires. *Journal of pediatric psychology*, *22*(3), 313-328. |
| MacArthur-Bates Communicative Development Inventories SL Adaptation (CDI) | 0-2.9 | Caregiver | Fenson, L. (2007). *MacArthur-Bates communicative development inventories*. Baltimore, MD: Paul H. Brookes Publishing Company. |
| Child Behavior Checklist (CBCL) | 0-2.9 | Caregiver | Achenbach, T.M., & Rescorla, L.A. (2000). *Manual for the ASEBA Preschool forms and Profiles.* Burlington, VT: University of Vermont Department of Psychiatry.  Achenbach, T.M., & Rescorla, L. A. (2001). *Manual for the ASEBA School-Age Forms and Profiles.* Burlington, VT: University of Vermont, Research Center for Children, Youth, and Families.  Achenbach, T. M., & Ruffle, T. M. (2000). The child behavior checklist and related forms for assessing behavioral/emotional problems and competencies. Pediatrics in Review, 21, 265–271. |
| Home Observation For Measurement of the Home Environment (HOME-South Africa) | 3-6.9 | Observer | Bradley, R.F., Corwyn, R. F., & Whiteside-Mansell. (1996). Life at Home: Same time, different places- an examination of the HOME Inventory in different cultures. *Early Development and Parenting*, 5(*4*) 251-269.  Caldwell, B. M. & Bradley, R. H. (2001). *HOME Inventory Administration Manual*. Little Rock: University of Arkansas at Little Rock. |
| Observation of Mother-Child Interaction (OMCI) | 3-6.9 | Observer | Rasheed, M.A., & Yousafzai, A.K. (2013 September). The development and reliability testing of an observation tool for assessing mother-child interaction in field studies- Experience from Pakistan In: The 16th European Conference on Developmental Psychology, Lausanne, Switzerland |
| Malawi Development Assessment Tool (MDAT) | 3-6.9 | Observer | Gladstone, M., Lancaster, G. A., Umar, E., Nyirenda, M., Kayira, E., van den Broek, N. R., & Smyth, R. L. (2010). The Malawi Developmental Assessment Tool (MDAT): the creation, validation, and reliability of a tool to assess child development in rural African settings. *PLoS Med*, *7*(5), e1000273. |
| Preschool Self-Regulation Assessment (PSRA) | 3-6.9 | Observer | Smith-Donald, R., Raver, C. C., Hayes, T., & Richardson, B. (2007). Preliminary construct and concurrent validity of the Preschool Self-regulation Assessment (PSRA) for field-based research. *Early Childhood Research Quarterly*, *22*(2), 173–187. <https://doi.org/10.1016/j.ecresq.2007.01.002> |
| The Early Grade Reading Assessment (EGRA) | 3-6.9 | Observer | ACER (March 2014). The Early Grade Reading Assessment: Assessing children's acquisition of basic literacy skills in developing countries. Assessment GEMs no.2. Melbourne: ACER |
| The Early Grade Math Assessment (EGMA) | 3-6.9 | Observer | ACER (March 2014). The Early Grade Reading Assessment: Assessing children's acquisition of basic literacy skills in developing countries. Assessment GEMs no.2. Melbourne: ACER |
| Parental Acceptance and Rejection Questionnaire Short Form (PARQ) | 3-6.9 | Caregiver | Rohner, R. P., & Khaleque, A. (Eds.). (2005). Handbook for the Study of Parental Acceptance and Rejection (4th ed). Storrs, CT: Rohner Research Publications. |
| Adapted UNICEF Multiple Indicator Cluster Survey (MICS) | 3-6.9 | Caregiver | UNICEF. Child Disciplinary Practices at Home. Retrieved July 16, 2020, from <https://www.unicef.org/protection/Child_Disciplinary_Practices_at_Home.pdf> |
| Ages and Stages Questionnaire (ASQ) | 3-6.9 | Caregiver | American Academy of Pediatrics. (2001). Developmental Surveillance and Screening of Infants and Young Children. *Pediatrics, 108*(1), 192–196.  American Academy of Pediatrics. (2006). Identifying Infants and Young Children with Developmental Disorders in the Medical Home: An Algorithm for Developmental Surveillance and Screening. *Pediatrics, 118*, 405–420.  Beam, M., Paré, E., Schellenbach, C., Kaiser, A., Murphy, M., (2015). Early Developmental Screeinng in High-Risk Communities: Implications for Research and Child Welfare Policy. *The Advanced Generalist: Social Work Research Journal, 1(3/4),* 18-3  Hanig, K. M. (2010). Review of Ages & Stages Questionnaires®: A Parent-Completed Child Monitoring System. In R. A. Spies, J. F. Carlson, & K. F. Geisinger (Eds.), *The eighteenth mental measurements yearbook,* 10–13. Lincoln, NE: Buros Institute of Mental Measurements. |
| Ages and Stages Questionnaire Social and Emotional (ASQ - SE) | 3-6.9 | Caregiver | Squires, J., Bricker, D., & Twombly, E. (2002). *The ASQ:SE user’s guide.* Baltimore, MD: Paul H. Brookes Publishing Co. |
| Child Behavior Checklist (CBCL) | 3-6.9 | Caregiver | Achenbach, T.M., & Rescorla, L.A. (2000). *Manual for the ASEBA Preschool forms and Profiles.* Burlington, VT: University of Vermont Department of Psychiatry.  Achenbach, T.M., & Rescorla, L. A. (2001). *Manual for the ASEBA School-Age Forms and Profiles.* Burlington, VT: University of Vermont, Research Center for Children, Youth, and Families.  Achenbach, T. M., & Ruffle, T. M. (2000). The child behavior checklist and related forms for assessing behavioral/emotional problems and competencies. Pediatrics in Review, 21, 265–271. |
| Devereux Early Childhood Assessment (DECA) | 3-6.9 | Caregiver | LeBuffe, P. A., & Naglieri, J. A. (1999). *Technical manual for the Devereux Early Childhood Assessment (DECA).* Villanova, PA: Devereux Foundation. |
| Normative Beliefs about Aggression Scale  (Youth Report) | 3-6.9 | Caregiver | Shahid, A., Wilkinson, K., Marcu, S., & Shapiro, C. M. (2011). Normative Beliefs About Aggression Scale. In STOP, THAT and One Hundred Other Sleep Scales (pp. 249-250). Springer New York. |
| The Early Grade Reading Assessment (EGRA) | 7+ | Observer | ACER (March 2014). The Early Grade Reading Assessment: Assessing children's acquisition of basic literacy skills in developing countries. Assessment GEMs no.2. Melbourne: ACER |
| The Early Grade Math Assessment (EGMA) | 7+ | Observer | ACER (March 2014). The Early Grade Reading Assessment: Assessing children's acquisition of basic literacy skills in developing countries. Assessment GEMs no.2. Melbourne: ACER |
| Parental Acceptance and Rejection Questionnaire Short Form (PARQ) | 7+ | Caregiver | Rohner, R. P., & Khaleque, A. (Eds.). (2005). Handbook for the Study of Parental Acceptance and Rejection (4th ed). Storrs, CT: Rohner Research Publications. |
| Adapted UNICEF Multiple Indicator Cluster Survey (MICS) | 7+ | Caregiver | UNICEF. Child Disciplinary Practices at Home. Retrieved July 16, 2020, from <https://www.unicef.org/protection/Child_Disciplinary_Practices_at_Home.pdf> |
| Child Behavior Checklist (CBCL) | 7+ | Caregiver | Achenbach, T.M., & Rescorla, L.A. (2000). *Manual for the ASEBA Preschool forms and Profiles.* Burlington, VT: University of Vermont Department of Psychiatry.  Achenbach, T.M., & Rescorla, L. A. (2001). *Manual for the ASEBA School-Age Forms and Profiles.* Burlington, VT: University of Vermont, Research Center for Children, Youth, and Families.  Achenbach, T. M., & Ruffle, T. M. (2000). The child behavior checklist and related forms for assessing behavioral/emotional problems and competencies. Pediatrics in Review, 21, 265–271. |
| Devereux Early Childhood Assessment (DECA) | 7+ | Caregiver | LeBuffe, P. A., & Naglieri, J. A. (1999). *Technical manual for the Devereux Early Childhood Assessment (DECA).* Villanova, PA: Devereux Foundation. |
| Oxford Measure of Psychosocial Adjustment (OMPA) | 7+ | Caregiver | MacMullin, C., Loughry, M. (2004). Investigating psychosocial adjustment of former child soldiers in Sierra Leone and Uganda. *Journal of Refugee Studies.*;17(4):460-472. |
| Emotional regulation (ERC) | 7+ | Caregiver | Shields, A. M., & Cicchetti, D. (1995, March). The development of an emotion regulation assessment battery: Reliability and validity among at-risk grade-school children. Poster session presented at the biennial meeting of the Society for Research in Child Development, Indianapo-lis, IN. |
| Normative Beliefs about Aggression Scale | 7+ | Self-report | Huesmann, L. R., & Guerra, N. G. (1997). Children's normative beliefs about aggression and aggressive behavior. Journal of Personality and Social Psychology, 72(2), 408-419. |
| World Health Organization Disability Assessment Schedule (WHO DAS) | 7+ | Self-report | WHO Disability Assessment Schedule 2.0 WHODAS 2.0. Retrieved July 16, 2020, from <https://www.who.int/classifications/icf/whodasii/en/index4.html> |
| Difficulties in Emotion Regulation Scale (DERS) | 7+ | Self-report | Gratz, K. L., & Roemer, L. (2004). Multidimensional assessment of emotion regulation and dysregulation: Development, factor structure, and initial validation of the difficulties in emotion regulation scale. *Journal of psychopathology and behavioral assessment*, *26*(1), 41-54. |
| Parental Acceptance-Rejection Questionnaire (PARQ) | 7+ | Self-report | Rohner, R. P., & Khaleque, A. (Eds.). (2005). Handbook for the Study of Parental Acceptance and Rejection (4th ed). Storrs, CT: Rohner Research Publications. |
| Oxford Measure of Psychosocial Adjustment (OMPA) | 7+ | Self-report | MacMullin, C., Loughry, M. (2004). Investigating psychosocial adjustment of former child soldiers in Sierra Leone and Uganda. *Journal of Refugee Studies.*;17(4):460-472. |
