# Supplementary material for: The intergenerational impact of war on mental health and psychosocial wellbeing: lessons from the longitudinal study of war-affected youth in Sierra Leone
Source: Confl Health. 2020 Sep 1;14:62. doi: 10.1186/s13031-020-00308-7 (PMC7461150; doi:10.1186/s13031-020-00308-7)
Supplement: Supplementary file 2 — Additional file 2. EVD Measures. [file 13031_2020_308_MOESM2_ESM.docx]

**Appendix B. EVD Measures**

| **Measure** | **Source** |
| --- | --- |
| Physical Health Status | Cozma, V. S. (2014). Attitudes and Practices (KAP) Survey on the Ebola Virus Disease (EVD)—Kailahun and Kenema Districts, Sierra Leone. |
| Media Exposure to Ebola Messaging | National Institute of Drug Abuse. National Survey of Parents and Youth Wave 4. 2002. Retrieved from <https://archives.drugabuse.gov/sites/default/files/survey-wave4_1.pdf> |
| Information and Health Seeking Behaviors Related to Ebola | Self-created, cited here: Betancourt TS, Brennan RT, Vinck P, VanderWeele TJ, Spencer-Walters D, Jeong J, Akinsulure-Smith A, Pham P. Associations between Mental Health and Ebola-Related Health Behaviors: A Regionally Representative Cross-sectional Survey in Postconflict Sierra Leone. PLoS Med. 2016; 13(8): e1002073. |
| Changes in behavior as a result of the Ebola epidemic |  |
| Beliefs About Ebola | Duncan, L. A., Schaller, M., & Park, J. H. (2009). Perceived vulnerability to disease: Development and validation of a 15-item self-report instrument. *Personality and Individual differences*, *47*(6), 541-546. |
| Personal Exposure to Ebola Virus Disease | Self-created, cited here: Betancourt TS, Brennan RT, Vinck P, VanderWeele TJ, Spencer-Walters D, Jeong J, Akinsulure-Smith A, Pham P. Associations between Mental Health and Ebola-Related Health Behaviors: A Regionally Representative Cross-sectional Survey in Postconflict Sierra Leone. PLoS Med. 2016; 13(8): e1002073. |
| Approval of response agencies to Ebola epidemic |  |
| Perception of risk to contract Ebola virus disease | de Zwart, O., Veldhuijzen, I. K., Elam, G., Aro, A. R., Abraham, T., Bishop, G. D., Voeten, H. A., Richardus, J. H., & Brug, J. (2009). Perceived threat, risk perception, and efficacy beliefs related to SARS and other (emerging) infectious diseases: results of an international survey. International Journal of Behavioral Medicine, 16(1), 30–40. |
| Adult Mental Health and Adjustment | Betancourt TS, Borisova II, de la Soudière M, Williamson J. Sierra Leone’s child soldiers: war exposures and mental health problems by gender. J Adolesc Health. 2011; 49:21–28.  Derogatis LR, Lipman RS, Rickels K, Uhlenhuth EH, Covi L. The Hopkins Symptom Checklist (HSCL): a self-report symptom inventory. Behav Sci. 1974; 19:1–15. |
| Mental Health | Johnson K, Asher J, Rosborough S, Raja A, Panjabi R, Beadling C, et al. Association of combatant status and sexual violence with health and mental health outcomes in postconflict Liberia. JAMA. 2008; 300:676–690. |
| Post War Hardships and Daily Hassles | Layne, C.M., Stuvland, R., Saltzman, W., Djapo, N., & Pynoos, R.S. (1999). *Adolescent Post War Adversities Scale*: Unpublished instrument. |
| Health Status | UNICEF. Study on Public Knowledge, Attitudes, and Practices Relating to Ebola Virus Disease (EVD) Prevention and Medical Care in Sierra Leone. 2014. Retrieved from <https://reliefweb.int/sites/reliefweb.int/files/resources/Ebola-Virus-Disease-National-KAP-Study-Final-Report_-final.pdf> |
| Personal and Institutional Efficacy | UNICEF. Study on Public Knowledge, Attitudes, and Practices Relating to Ebola Virus Disease (EVD) Prevention and Medical Care in Sierra Leone. 2014. Retrieved from <https://reliefweb.int/sites/reliefweb.int/files/resources/Ebola-Virus-Disease-National-KAP-Study-Final-Report_-final.pdf> |
| Stigma towards Ebola survivors | Kalichman, S. C., Simbayi, L. C., Jooste, S., Toefy, Y., Cain, D., Cherry, C., & Kagee, A. (2005). Development of a brief scale to measure AIDS-related stigma in South Africa. AIDS and Behavior, 9(2), 135-143.  Uys, L. R., Holzemer, W. L., Chirwa, M. L., Dlamini, P. S., Greeff, M., Kohi, T. W., … Naidoo, J. R. (2009). The development and validation of the HIV/AIDS Stigma Instrument - Nurse (HASI-N). AIDS care, 21(2), 150–159. |
